# Supplementary material for: Using country-level variables to discover country clusters beyond traditional health policy and performance metrics: An unsupervised machine learning approach for HIV healthcare delivery and financing
Source: PLOS Glob Public Health. 2025 Dec 2;5(12):e0004583. doi: 10.1371/journal.pgph.0004583 (PMC12671754; doi:10.1371/journal.pgph.0004583)
Supplement: S1 Text — Supporting information for Using country-level variables to discover country clusters beyond traditional health policy and performance metrics: An unsupervised machine learning approach for HIV healthcare delivery and financing. (DOCX) [file pgph.0004583.s001.docx]

**S1 Text. Supporting information.** Supporting information for *Using country-level variables to discover country clusters beyond traditional health policy and performance metrics: An unsupervised machine learning approach for HIV healthcare delivery and financing.*

**Text A**

**Data selection and pre-processing**

The data was sourced from various databases like WDI-World Bank, DHS, UNAIDS, WHO Global Health Observatory, National Commitments and Policy Instrument etc. Many variables had the problem of missing data, and to manage data missingness we adopted the following strategies:

- In consultation with the study team, we included data for the years for which majority of the countries had most data available for a given variable. For example, the variable for life expectancy at birth, for both female and male had a lot of missing data for many countries for the latest years of 2019 and later. Therefore, instead of using 2019 data, we included 2017 data for this variable in our database. Similar, procedure was followed for some other variables with lot of missing data for recent years for most countries.
- DHS surveys are not conducted each year in every DHS program country. Also, these surveys are not simultaneously undertaken in every DHS program country. Furthermore, there are some countries where the surveys were conducted more frequently than other countries. These varied surveys have resulted in data for some countries to be available for many times in our data extraction window starting in 2013, and for some countries the data was only available once in this window. Also, there were many countries in the DHS program which did not have any data in this window. To handle this issue of both irregular and multiple data availability for different years for the DHS countries, we decided to take the average of all the data available for every DHS country from 2013 to 2019. This helped us obtain a single data point and also reduced some amount of missing information for the DHS variables.
- Even after adopting different methodologies to reduce the amount of missing data in the database, many variables still had missing information. Therefore, before running the analysis we decided to drop variables which had more than 75% of missing data.

In addition to handing the missing information in our database, we created some compound variables. We created variables by taking ratios of the base variables downloaded from open-source databases. For example, we included a variable called “People using at least basic sanitation services (% of population): Q1/Q5” which is the ratio of People using at least basic sanitation services (% of population): Q1” to “People using at least basic sanitation services (% of population): Q5”.

Similar methodology was followed to create other variables which we included in our database as presented in **Table A**.

The following table shows the missingness per variable before dropping the variables which had more than 75% of missing data.

**Table A: Variables which we included in our database, with missing counts and percentages.**

| **Variable** | **Missing count** | **Missingness (%)** |
| --- | --- | --- |
| TotalPrivate ratio Prevention of vertical transmission of HIV (subtotal) | 106 | 79.10 |
| GDP/cap growth 2000-2018 (WHO) | 102 | 76.12 |
| HIV prevalence transgender people 2019 | 101 | 75.37 |
| Implementation of ART provision in community settings for people stable on ART | 96 | 71.64 |
| hypertensive,n (%) | 94 | 70.15 |
| Benefits package for universal health insurance scheme includes ARVs (for countries with such a scheme) | 93 | 69.40 |
| budget trend 20-21 | 93 | 69.40 |
| TotalPrivate ratio Treatment, care and support | 92 | 68.66 |
| TotalPrivate ratio Prevention | 91 | 67.91 |
| Gini index (World Bank estimate) | 90 | 67.16 |
| Children with ARI for whom advice or treatment was sought from a health facility or provider DHS 2013-19 | 87 | 64.93 |
| Received all 8 basic vaccinations DHS 2013-19 | 86 | 64.18 |
| Under-five mortality rate DHS 2013-19 | 86 | 64.18 |
| 2020 prevention targets for MSM: % of MSM reached by HIV prevention services | 86 | 64.18 |
| Implementation status of same day start of ART | 86 | 64.18 |
| International ratio Prevention of vertical transmission of HIV (subtotal) | 86 | 64.18 |
| WHO 2015 policy update on use of LF-LAM for diagnosis and screening of active TB in people living with HIV adopted | 85 | 63.43 |
| Unmet need for family planning DHS 2013-19 | 84 | 62.69 |
| 2020 prevention targets for sex workers: % of sex workers reached by HIV prevention services | 84 | 62.69 |
| Total public ratio prevention of vertical transmission of HIV (subtotal) | 84 | 62.69 |
| Prevalence of diabetes mellitus (%) | 81 | 60.45 |
| HIV prevalence PWID 2019 | 80 | 59.70 |
| Self-testing implemented (in countries with a national self-testing policy) | 79 | 58.96 |
| Universal health insurance scheme in country | 76 | 56.72 |
| Total public ratio Prevention | 75 | 55.97 |
| Routine user fees or charges for services at public health facilities | 74 | 55.22 |
| International ratio Prevention | 71 | 52.99 |
| HIV case surveillance system in the country | 69 | 51.49 |
| Compulsory detention for people who use drugs applied | 69 | 51.49 |
| International ratio Treatment, care and support | 64 | 47.76 |
| Catastrophic spending (% of population spending >10% of income on OOP) | 63 | 47.01 |
| Any laws/provisions specifying protections based on grounds of sexual orientation | 63 | 47.01 |
| HIV expenditure on key populations (aggregate) (% of all HIV exp. in year) | 62 | 46.27 |
| HIV prevalence prisoners 2019 | 62 | 46.27 |
| Total public ratio treatment, care and support | 60 | 44.78 |
| Any restrictions to registration/operation of civil society/CBOs affecting HIV service delivery | 58 | 43.28 |
| Method to de-duplicate key data (i.e. unique identifiers) | 52 | 38.81 |
| Total expenditures on HIV (USD) | 49 | 36.57 |
| Possible to start ART on the same day as HIV diagnosis | 48 | 35.82 |
| Health facilities delivering integrated services: Violence screening and mitigation with HIV services | 47 | 35.07 |
| Antiretroviral therapy coverage for PMTCT (% of pregnant women living with HIV) | 46 | 34.33 |
| Health facilities delivering integrated services: HIV and hepatitis C treatment | 46 | 34.33 |
| UserFee NoUse PHC HIV | 46 | 34.33 |
| Health facilities delivering integrated services: ART and chronic NCDs | 45 | 33.58 |
| Health facilities delivering integrated services: ART and general outpatient care | 45 | 33.58 |
| Health facilities delivering integrated services: HIV and harm reduction services | 45 | 33.58 |
| Health facilities delivering integrated services: HIV testing with child health services | 45 | 33.58 |
| Explicit supportive reference to harm reduction in national policies | 45 | 33.58 |
| Health facilities delivering integrated services: HIV counselling and testing and chronic NCDs | 44 | 32.84 |
| Health facilities delivering integrated services: HIV testing and counselling with cervical cancer screening and treatment | 44 | 32.84 |
| Health facilities delivering integrated services: HIV treatment and care with child health services | 44 | 32.84 |
| Health facilities delivering integrated services: HIV treatment and care with nutrition support | 44 | 32.84 |
| Human rights monitoring and enforcement mechanisms: Independent functional institution for promotion and protection of human rights | 44 | 32.84 |
| National plan/strategy addressing gender-based violence/violence against women that includes HIV | 44 | 32.84 |
| WHO recommendation on oral PrEP adopted in country's national guidelines | 44 | 32.84 |
| Health facilities delivering integrated services: HIV counselling and testing and general outpatient care | 43 | 32.09 |
| People using at least basic sanitation services (% of population): Q1/Q5 | 42 | 31.34 |
| Health facilities delivering integrated services: Cervical cancer screening with HIV services | 42 | 31.34 |
| Health facilities delivering integrated services: PMTCT with antenatal care / maternal and child health | 42 | 31.34 |
| Education policies on life skills-based HIV and sexuality education - primary school | 42 | 31.34 |
| Prevalence of current tobacco use (% of adults) | 41 | 30.60 |
| Health facilities delivering integrated services: ART and TB treatment | 41 | 30.60 |
| Health facilities delivering integrated services: HIV counselling and testing with SRH | 41 | 30.60 |
| Health facilities delivering integrated services: HIV counselling and testing with TB services | 41 | 30.60 |
| Health facilities delivering integrated services: HIV treatment and care with SRH | 41 | 30.60 |
| Health facilities delivering integrated services: TB screening in HIV services | 41 | 30.60 |
| Total wealth per capita (constant 2014 US$), 2014 | 38 | 28.36 |
| HIV prevalence - Adults (15-49) | 37 | 27.61 |
| Laws criminalizing transmission of non-disclosure of or exposure to HIV transmission | 37 | 27.61 |
| Global Fund resources | 36 | 26.87 |
| Criminalization of sex work | 36 | 26.87 |
| Laws requiring parental consent for adolescents to access HIV testing | 36 | 26.87 |
| New HIV infections (all ages) | 34 | 25.37 |
| ART provided in community settings for people stable on ART | 34 | 25.37 |
| Country has a national plan for eliminating HIV MTCT | 34 | 25.37 |
| Nurse-initiated ART allowed for non-pregnant adults | 33 | 24.63 |
| Number of people living with HIV (all ages) | 31 | 23.13 |
| HIV prevalence MSM 2019 | 30 | 22.39 |
| Antiretroviral therapy coverage (% of people living with HIV) | 27 | 20.15 |
| Assisted HIV partner notification included in national policy | 27 | 20.15 |
| National policy on routine viral load testing: for adults and adolescents | 24 | 17.91 |
| HIV prevalence sex workers 2019 | 23 | 17.16 |
| DTG being introduced as first-line ARV regimen (where TDF/3TC or (FTC)/EFV not preferred combinations) | 21 | 15.67 |
| Recommended ART CD4 initiation threshold in adults and adolescents | 21 | 15.67 |
| Country has a national policy on HIV self-testing | 21 | 15.67 |
| Criminalization of same sex sexual acts | 19 | 14.18 |
| income share: Top10% 2019 | 17 | 12.69 |
| Income share: bottom50% 2019 | 17 | 12.69 |
| COVID case fatality ratio | 16 | 11.94 |
| Hospital beds (per 10 000 population) | 13 | 9.70 |
| Debt to GDP ratio 2015 | 11 | 8.21 |
| Pharmacists (per 10,000) | 11 | 8.21 |
| GDP per capita, PPP (current international $) | 8 | 5.97 |
| Year dentists (per 10,000) | 7 | 5.22 |
| Current health expenditure (% of GDP) | 6 | 4.48 |
| Domestic general government health expenditure (% of current health expenditure) | 6 | 4.48 |
| External health expenditure (% of current health expenditure) | 6 | 4.48 |
| Out-of-pocket expenditure (% of current health expenditure) | 6 | 4.48 |
| Physicians’ density (per 1000 population) | 6 | 4.48 |
| Nursing and midwifery personnel density (per 1000 population) | 6 | 4.48 |
| Population ages 0-14 (% of total population) | 5 | 3.73 |
| Population ages 15-64 (% of total population) | 5 | 3.73 |
| Population ages 65 and above (% of total population) | 5 | 3.73 |
| Tuberculosis effective treatment coverage (%) | 5 | 3.73 |
| Life expectancy at birth, total (years) | 4 | 2.99 |
| Life expectancy at age 60, female (years) 2017 | 4 | 2.99 |
| Life expectancy at age 60, male (years) 2017 | 4 | 2.99 |
| Maternal mortality ratio (modeled estimate, per 100,000 live births) | 4 | 2.99 |
| Immunization, DPT (% of children ages 12-23 months) | 2 | 1.49 |
| Tuberculosis case detection rate (%, all forms) | 2 | 1.49 |
| Tuberculosis treatment success rate (% of new cases) | 2 | 1.49 |
| Urban population (% of total population) | 1 | 0.75 |
| Voice and Accountability: Percentile Rank | 1 | 0.75 |

**Fig A. Gap statistic for Partitioning Around Medoids clustering algorithm.** Validation and robustness check for the algorithm.


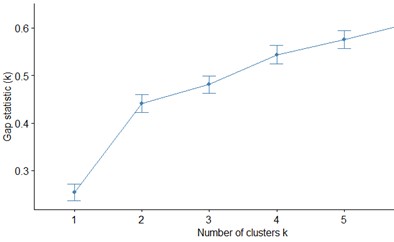


**Table B. Adjusted Rand Index (ARI) values for comparison across four cluster algorithms.**

|  | **PAM** | **HC** | **DBSCAN** | **Spectral** |
| --- | --- | --- | --- | --- |
| PAM | 1 | 0.929 | 0.71 | 0.835 |
| HC | .. | 1 | 0.672 | 0.817 |
| DBSCAN | .. | .. | 1 | 0.666 |
| Spectral | .. | .. | .. | 1 |

PAM (Partitioning Around Medoids), HC (Hierarchical Clustering), DBSCAN (Density-Based Spatial Clustering of Applications with Noise)

**Fig B. Country maps for A) HC (Hierarchical Clustering), B) DBSCAN (Density-Based Spatial Clustering of Applications with Noise) and C) Spectral Clustering.** Source of the basemap shapefile: a SpatialPolygonsDataFrame object containing a simplified world map with polygons attributed to 244 countries, based on Natural Earth data (© Natural Earth, www.naturalearthdata.com), public domain.

A.


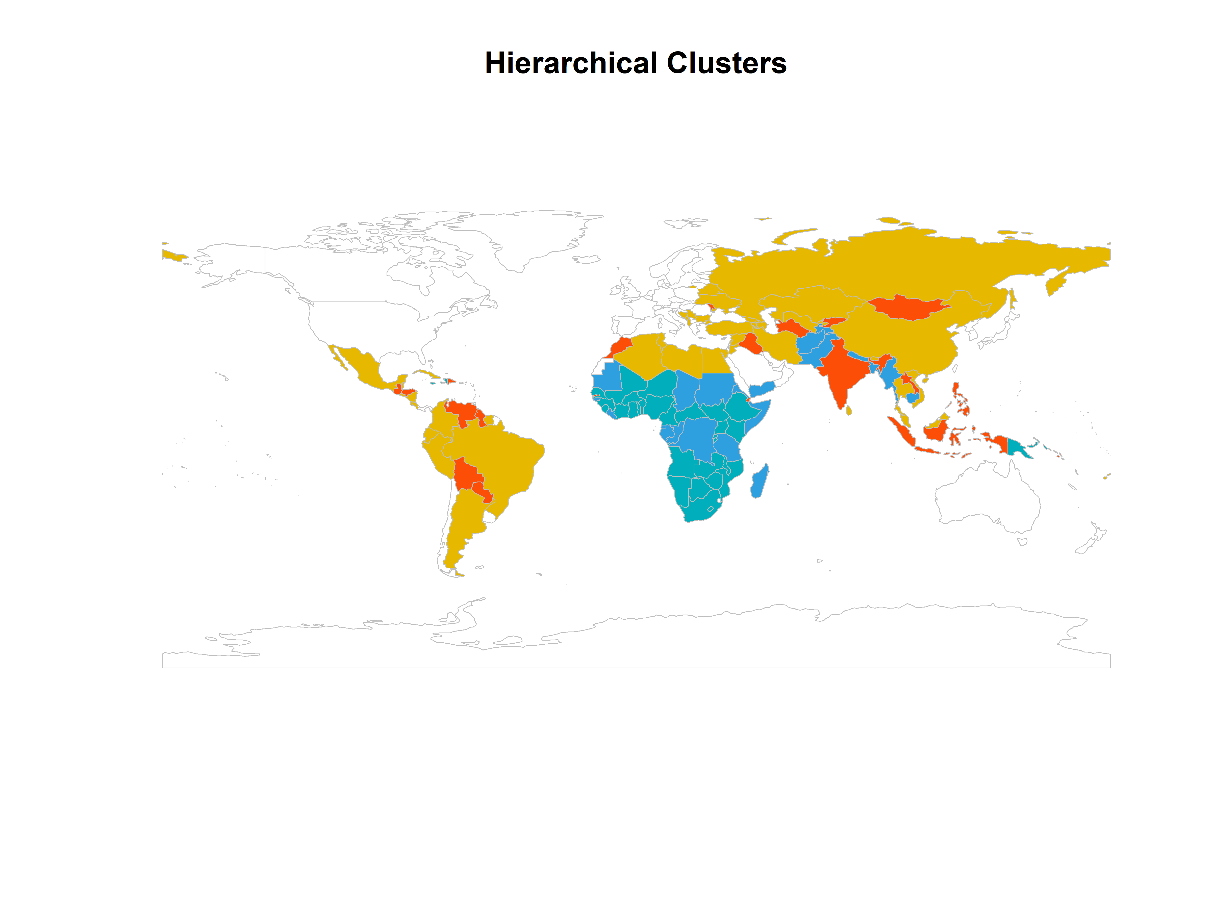


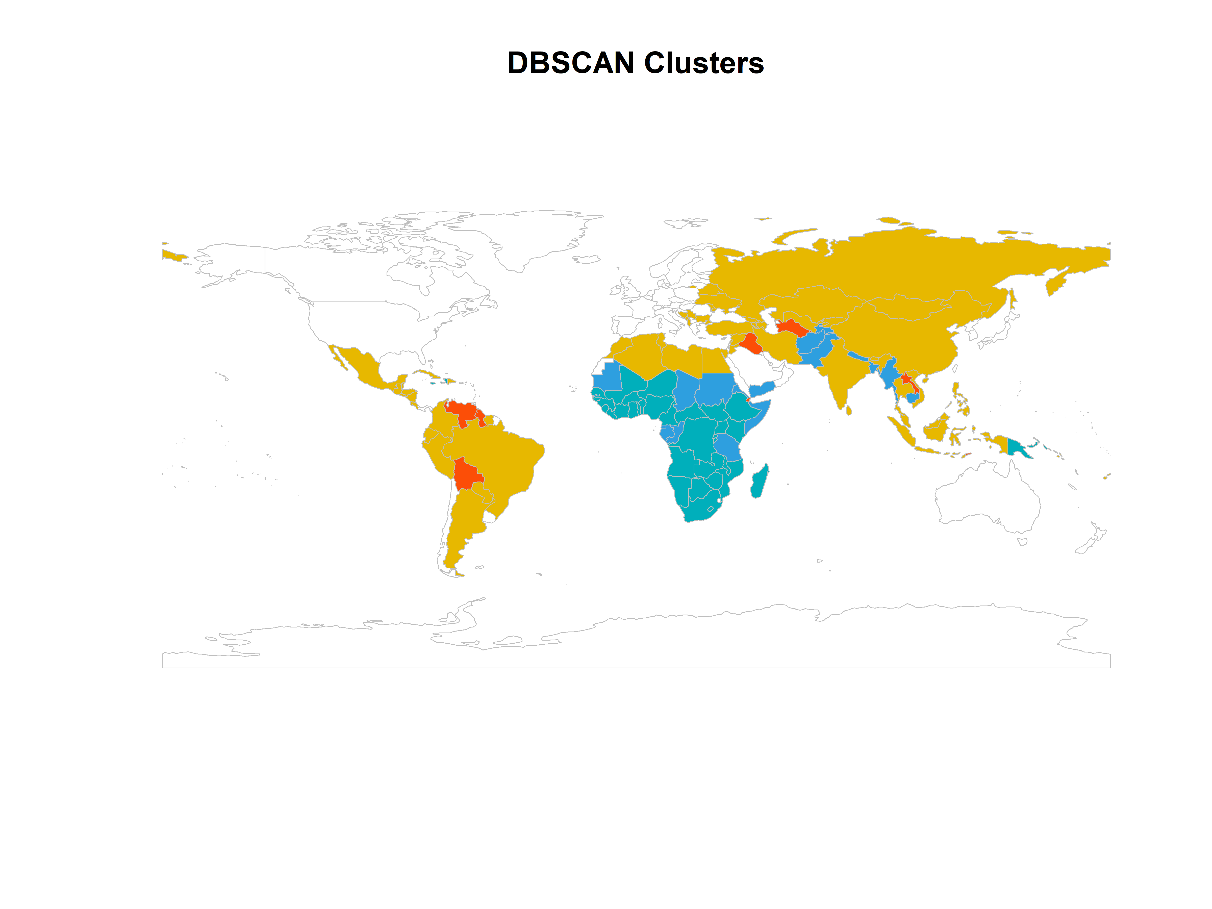
B.


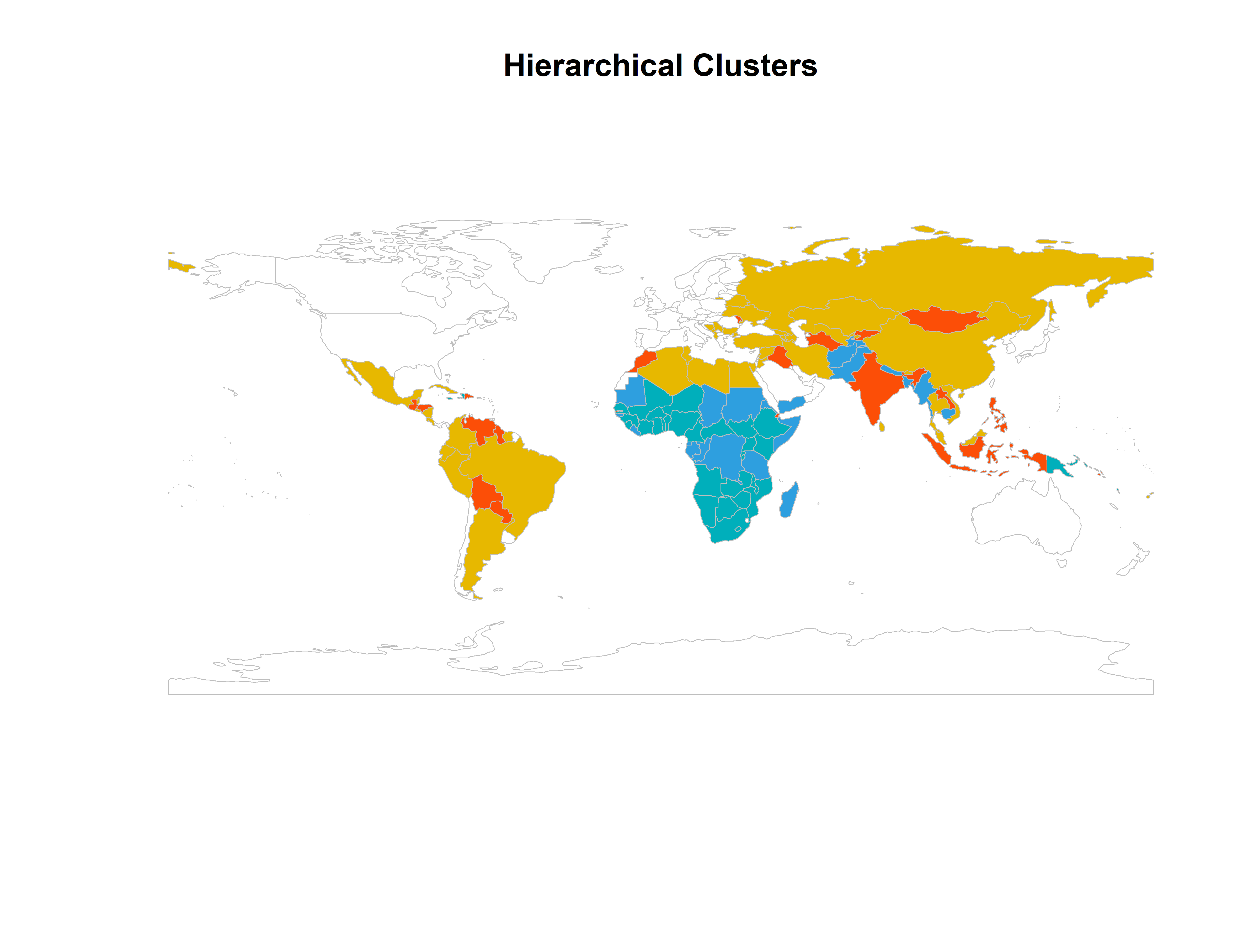
C.

**Table C. Country clusters as computed by PAM (Partitioning Around Medoids).**

| **Cluster 1** | **Cluster 2** | **Cluster 3** | **Cluster 4** |
| --- | --- | --- | --- |
| Afghanistan | Burkina Faso | Korea, Dem. People's Rep. | Bolivia |
| Chad | Burundi | Syrian Arab Republic | Cabo Verde |
| Eritrea | Central African Republic | Algeria | Djibouti |
| Gambia, The | Congo, Dem. Rep. | Bhutan | Honduras |
| Madagascar | Ethiopia | Egypt, Arab Rep. | India |
| Somalia | Guinea | Nicaragua | Kiribati |
| Sudan | Guinea-Bissau | Sri Lanka | Kyrgyz Republic |
| Tajikistan | Haiti | Tunisia | Lao PDR |
| Yemen, Rep. | Liberia | Ukraine | Micronesia, Fed. Sts. |
| Bangladesh | Malawi | Uzbekistan | Moldova |
| Cambodia | Mali | Albania | Mongolia |
| Comoros | Mozambique | American Samoa | Morocco |
| Congo, Rep. | Niger | Argentina | Philippines |
| Myanmar | Rwanda | Armenia | Timor-Leste |
| Nepal | Sierra Leone | Azerbaijan | Marshall Islands |
| Pakistan | South Sudan | Belarus | Guatemala |
| Tanzania | Togo | Belize | Guyana |
| Equatorial Guinea | Uganda | Bosnia and Herzegovina | Indonesia |
| Mauritania | Vanuatu | Brazil | Iraq |
|  | Eswatini | Bulgaria | Paraguay |
|  | Ghana | China | Samoa |
|  | Kenya | Colombia | Tonga |
|  | Lesotho | Costa Rica | Turkmenistan |
|  | Nigeria | Cuba | Venezuela, RB |
|  | Sao Tome and Principe | Ecuador |  |
|  | Senegal | Fiji |  |
|  | Zambia | Georgia |  |
|  | Zimbabwe | Iran, Islamic Rep. |  |
|  | Angola | Jordan |  |
|  | Botswana | Lebanon |  |
|  | Gabon | Libya |  |
|  | Jamaica | Malaysia |  |
|  | Namibia | Maldives |  |
|  | Papua New Guinea | Mexico |  |
|  | South Africa | Montenegro |  |
|  | Vanuatu | North Macedonia |  |
|  |  | Serbia |  |
|  |  | St. Lucia |  |
|  |  | St. Vincent and the Grenadines |  |
|  |  | Suriname |  |
|  |  | Thailand |  |
|  |  | Turkey |  |
|  |  | Tuvalu |  |

**Table D. Country clusters as computed by HC (Hierarchical Clustering).**

| **Cluster 1** | **Cluster 2** | **Cluster 3** | **Cluster 4** |
| --- | --- | --- | --- |
| Afghanistan | Burkina Faso | Gambia, The | Korea, Dem. People's Rep. |
| Chad | Burundi | Bolivia | Syrian Arab Republic |
| Congo, Dem. Rep. | Central African Republic | Cabo Verde | Algeria |
| Eritrea | Ethiopia | Djibouti | Bhutan |
| Guinea-Bissau | Guinea | Honduras | Egypt, Arab Rep. |
| Liberia | Haiti | India | El Salvador |
| Madagascar | Malawi | Kiribati | Nicaragua |
| Somalia | Mali | Lao PDR | Sri Lanka |
| Sudan | Mozambique | Micronesia, Fed. Sts. | Tunisia |
| Tajikistan | Niger | Moldova | Ukraine |
| Yemen, Rep. | Rwanda | Mongolia | Uzbekistan |
| Bangladesh | Sierra Leone | Morocco | Albania |
| Cambodia | South Sudan | Philippines | American Samoa |
| Comoros | Togo | Solomon Islands | Argentina |
| Myanmar | Uganda | Timor-Leste | Armenia |
| Pakistan | Angola | Paraguay | Azerbaijan |
| Tanzania | Cameroon | Guyana | Belarus |
| Equatorial Guinea | Cote d'Ivoire | Indonesia | Belize |
| Gabon | Eswatini | Iraq | Bosnia and Herzegovina |
|  | Ghana | Marshall Islands | Brazil |
|  | Kenya | Samoa | Bulgaria |
|  | Nigeria | Tonga | China |
|  | Papua New Guinea | Venezuela, RB | Colombia |
|  | Sao Tome and Principe | Guatemala | Costa Rica |
|  | Senegal |  | Cuba |
|  | Vanuatu |  | Dominica |
|  | Zambia |  | Georgia |
|  | Zimbabwe |  | Grenada |
|  | Jamaica |  | Iran, Islamic Rep. |
|  | Namibia |  | Jordan |
|  | South Africa |  | Kazakhstan |
|  |  |  | Lebanon |
|  |  |  | Libya |
|  |  |  | Malaysia |
|  |  |  | Maldives |
|  |  |  | Mexico |
|  |  |  | Montenegro |
|  |  |  | North Macedonia |
|  |  |  | Peru |
|  |  |  | Russian Federation |
|  |  |  | Serbia |
|  |  |  | St. Lucia |
|  |  |  | St. Vincent and the Grenadines |
|  |  |  | Suriname |
|  |  |  | Thailand |
|  |  |  | Tuvalu |
|  |  |  | Turkey |

**Table E. Country clusters as computed by DBSCAN (Density-Based Spatial Clustering of Applications with Noise)**

| **Cluster 1** | **Cluster 2** | **Cluster 3** | **Cluster 4** |
| --- | --- | --- | --- |
| Afghanistan | Burkina Faso | Korea, Dem. People's Rep. | Bolivia |
| Chad | Burundi | Syrian Arab Republic | Cabo Verde |
| Eritrea | Central African Republic | Algeria | Lao PDR |
| Gambia, The | Congo, Dem. Rep. | Bhutan | Djibouti |
| Somalia | Ethiopia | Egypt, Arab Rep. | Timor-Leste |
| Sudan | Guinea | El Salvador | Guyana |
| Tajikistan | Guinea-Bissau | Honduras | Iraq |
| Yemen, Rep. | Haiti | India | Turkmenistan |
| Bangladesh | Liberia | Kyrgyz Republic | Venezuela, RB |
| Cambodia | Madagascar | Micronesia, Fed. Sts. |  |
| Comoros | Malawi | Moldova |  |
| Congo, Rep. | Mali | Mongolia |  |
| Mauritania | Mozambique | Morocco |  |
| Myanmar | Niger | Nicaragua |  |
| Nepal | Rwanda | Philippines |  |
| Pakistan | Sierra Leone | Solomon Islands |  |
| Tanzania | South Sudan | Sri Lanka |  |
|  | Togo | Tunisia |  |
|  | Uganda | Ukraine |  |
|  | Vanuatu | Uzbekistan |  |
|  | Zambia | Vietnam |  |
|  | Zimbabwe | West Bank and Gaza |  |
|  | Benin | Albania |  |
|  | Cameroon | American Samoa |  |
|  | Cote d'Ivoire | Argentina |  |
|  | Eswatini | Armenia |  |
|  | Ghana | Azerbaijan |  |
|  | Kenya | Belarus |  |
|  | Nigeria | Belize |  |
|  | Papua New Guinea | Bosnia and Herzegovina |  |
|  | Sao Tome and Principe | Brazil |  |
|  | Senegal | Bulgaria |  |
|  | Botswana | China |  |
|  | South Africa | Colombia |  |
|  | Namibia | Costa Rica |  |
|  | Jamaica | Cuba |  |
|  |  | Dominica |  |
|  |  | Dominican Republic |  |
|  |  | Ecuador |  |
|  |  | Georgia |  |
|  |  | Grenada |  |
|  |  | Guatemala |  |
|  |  | Indonesia |  |
|  |  | Iran, Islamic Rep. |  |
|  |  | Jordan |  |
|  |  | Kazakhstan |  |
|  |  | Lebanon |  |
|  |  | Libya |  |
|  |  | Malaysia |  |
|  |  | Maldives |  |
|  |  | Marshall Islands |  |
|  |  | Mexico |  |
|  |  | North Macedonia |  |
|  |  | Paraguay |  |
|  |  | Peru |  |
|  |  | Russian Federation |  |
|  |  | Samoa |  |
|  |  | Serbia |  |
|  |  | St. Lucia |  |
|  |  | St. Vincent and the Grenadines |  |
|  |  | Suriname |  |
|  |  | Thailand |  |
|  |  | Tonga |  |
|  |  | Turkey |  |
|  |  | Tuvalu |  |

**Table F. Country clusters as computed by Spectral Clustering.**

| **Cluster 1** | **Cluster 2** | **Cluster 3** | **Cluster 4** |
| --- | --- | --- | --- |
| Afghanistan | Burkina Faso | Korea, Dem. People's Rep. | Gambia, The |
| Chad | Burundi | Syrian Arab Republic | Bolivia |
| Eritrea | Central African Republic | Algeria | Cabo Verde |
| Somalia | Congo, Dem. Rep. | Bhutan | Djibouti |
| Tajikistan | Ethiopia | Egypt, Arab Rep. | El Salvador |
| Yemen, Rep. | Guinea | Nicaragua | Honduras |
| Bangladesh | Guinea-Bissau | Sri Lanka | India |
| Cambodia | Haiti | Tunisia | Kiribati |
| Comoros | Liberia | Ukraine | Lao PDR |
| Mauritania | Malawi | Vietnam | Micronesia, Fed. Sts. |
| Myanmar | Mali | West Bank and Gaza | Moldova |
| Pakistan | Mozambique | Albania | Mongolia |
| Tanzania | Niger | American Samoa | Morocco |
| Equatorial Guinea | Rwanda | Argentina | Philippines |
| Gabon | Sierra Leone | Armenia | Solomon Islands |
|  | South Sudan | Azerbaijan | Timor-Leste |
|  | Togo | Belarus | Guyana |
|  | Uganda | Belize | Indonesia |
|  | Angola | Bosnia and Herzegovina | Iraq |
|  | Cameroon | Brazil | Marshall Islands |
|  | Cote d'Ivoire | Bulgaria | North Macedonia |
|  | Eswatini | China | Paraguay |
|  | Ghana | Colombia | Peru |
|  | Kenya | Costa Rica | Samoa |
|  | Nigeria | Cuba | Serbia |
|  | Papua New Guinea | Dominica | Thailand |
|  | Sao Tome and Principe | Ecuador | Tonga |
|  | Senegal | Fiji | Marshall Islands |
|  | Vanuatu | Georgia | Turkey |
|  | Zambia | Grenada | Turkmenistan |
|  | Zimbabwe | Guatemala | Venezuela, RB |
|  | Jamaica | Iran, Islamic Rep. |  |
|  | Namibia | Jordan |  |
|  | South Africa | Kazakhstan |  |
|  |  | Lebanon |  |
|  |  | Libya |  |
|  |  | Malaysia |  |
|  |  | Maldives |  |
|  |  | Mexico |  |
|  |  | Russian Federation |  |
|  |  | St. Lucia |  |
|  |  | St. Vincent and the Grenadines |  |
|  |  | Suriname |  |
|  |  | Tuvalu |  |
|  |  | Turkey |  |

**Table G. Fuzzy PAM clustering membership probabilities.**

| **Country** | **Cluster 1** | **Cluster 2** | **Cluster 3** | **Cluster 4** |
| --- | --- | --- | --- | --- |
| Afghanistan | 72.2 | 14.2 | 5.1 | 8.5 |
| Burkina Faso | 23.1 | 65.3 | 4.7 | 6.9 |
| Burundi | 10.7 | 82.1 | 3 | 4.2 |
| Central African Republic | 12 | 80.7 | 3 | 4.3 |
| Chad | 75.2 | 9.7 | 5.3 | 9.8 |
| Congo, Dem. Rep. | 41.1 | 43.1 | 6.3 | 9.5 |
| Eritrea | 68.2 | 11.3 | 7.2 | 13.2 |
| Ethiopia | 21.9 | 67 | 4.6 | 6.6 |
| Gambia, The | 52.6 | 12.5 | 11.3 | 23.6 |
| Guinea | 15.5 | 74.9 | 4 | 5.6 |
| Guinea-Bissau | 37.3 | 47.3 | 6.2 | 9.2 |
| Haiti | 17.5 | 71.6 | 4.6 | 6.3 |
| Korea, Dem. People's Rep. | 7 | 4.4 | 70.9 | 17.8 |
| Liberia | 40.3 | 44.1 | 6.2 | 9.3 |
| Madagascar | 50 | 33.5 | 6.5 | 10 |
| Malawi | 13.4 | 76.3 | 4.4 | 5.9 |
| Mali | 26.1 | 60.4 | 5.6 | 8 |
| Mozambique | 11.3 | 81.5 | 3 | 4.2 |
| Niger | 16.1 | 74.8 | 3.7 | 5.3 |
| Rwanda | 15.9 | 71.3 | 5.4 | 7.4 |
| Sierra Leone | 22.5 | 64.3 | 5.5 | 7.7 |
| Somalia | 77.4 | 9.6 | 4.7 | 8.3 |
| South Sudan | 26.6 | 59 | 5.9 | 8.4 |
| Sudan | 74.4 | 10.5 | 5.4 | 9.7 |
| Syrian Arab Republic | 4.9 | 3 | 75.3 | 16.8 |
| Tajikistan | 63.9 | 21.2 | 5.7 | 9.2 |
| Togo | 22 | 65.4 | 5.2 | 7.4 |
| Uganda | 12.6 | 77.8 | 4 | 5.5 |
| Yemen, Rep. | 71.1 | 10.3 | 6.5 | 12.2 |
| Algeria | 9.3 | 5.4 | 36.4 | 48.9 |
| Angola | 11.1 | 81.4 | 3.1 | 4.3 |
| Bangladesh | 53.9 | 29.1 | 6.6 | 10.4 |
| Benin | 11.8 | 80.5 | 3.2 | 4.5 |
| Bhutan | 8.6 | 5.4 | 64.8 | 21.2 |
| Bolivia | 29.6 | 11 | 17.5 | 41.9 |
| Cabo Verde | 31.7 | 11.2 | 16.6 | 40.5 |
| Cambodia | 63.9 | 21 | 5.8 | 9.3 |
| Cameroon | 24.3 | 63 | 5.2 | 7.5 |
| Comoros | 73.8 | 10.9 | 5.6 | 9.7 |
| Congo, Rep. | 65.6 | 11.2 | 7.9 | 15.4 |
| Cote d'Ivoire | 11.7 | 80.3 | 3.3 | 4.7 |
| Djibouti | 41.6 | 12.4 | 14.1 | 31.9 |
| Egypt, Arab Rep. | 11.8 | 7.4 | 54.7 | 26.1 |
| El Salvador | 6.7 | 4 | 61.8 | 27.5 |
| Eswatini | 17.7 | 67.8 | 6.1 | 8.3 |
| Ghana | 21 | 65.8 | 5.5 | 7.7 |
| Honduras | 10.6 | 5 | 14.1 | 70.3 |
| India | 12 | 5.6 | 13.3 | 69.1 |
| Kenya | 14.7 | 73.6 | 4.9 | 6.7 |
| Kiribati | 12.4 | 5.7 | 13.1 | 68.8 |
| Kyrgyz Republic | 15.7 | 6.8 | 13.8 | 63.7 |
| Lao PDR | 36.7 | 11.5 | 14.7 | 37.1 |
| Lesotho | 16.5 | 69.9 | 5.8 | 7.8 |
| Mauritania | 74.8 | 9.7 | 5.5 | 10.1 |
| Micronesia, Fed. Sts. | 22.7 | 9 | 14.7 | 53.6 |
| Moldova | 9.9 | 5.2 | 18.8 | 66.1 |
| Mongolia | 10.1 | 4.9 | 13.8 | 71.3 |
| Morocco | 9.1 | 4.5 | 14.6 | 71.8 |
| Myanmar | 48 | 34.9 | 6.7 | 10.4 |
| Nepal | 52.1 | 31.6 | 6.4 | 10 |
| Nicaragua | 7.7 | 4.7 | 66.6 | 21 |
| Nigeria | 12.3 | 80.3 | 3.1 | 4.4 |
| Pakistan | 49.5 | 33.7 | 6.6 | 10.2 |
| Papua New Guinea | 12 | 79.5 | 3.5 | 4.9 |
| Philippines | 16.5 | 7.1 | 16.3 | 60.2 |
| Sao Tome and Principe | 15.2 | 74.4 | 4.3 | 6.1 |
| Senegal | 17.3 | 70.9 | 5 | 6.8 |
| Solomon Islands | 17.1 | 7.1 | 14.6 | 61.2 |
| Sri Lanka | 5 | 3.1 | 75.8 | 16 |
| Tanzania | 73.6 | 12.3 | 5.3 | 8.9 |
| Timor-Leste | 40.7 | 12 | 14.1 | 33.2 |
| Tunisia | 5 | 3.1 | 77.2 | 14.8 |
| Ukraine | 11.1 | 6.6 | 36.4 | 45.8 |
| Uzbekistan | 9.7 | 5.5 | 30.8 | 54 |
| Vanuatu | 20.9 | 65.4 | 5.8 | 8 |
| Vietnam | 10.7 | 6.7 | 57.7 | 24.9 |
| West Bank and Gaza | 4.7 | 2.9 | 78.2 | 14.2 |
| Zambia | 12.8 | 77.6 | 4.1 | 5.5 |
| Zimbabwe | 11.8 | 79.4 | 3.7 | 5.1 |
| Albania | 8.6 | 4.8 | 37.2 | 49.4 |
| American Samoa | 4.9 | 3 | 75.5 | 16.7 |
| Argentina | 6.9 | 4.4 | 68.8 | 19.9 |
| Armenia | 10.2 | 5.9 | 33.8 | 50 |
| Azerbaijan | 8.5 | 5 | 45.2 | 41.3 |
| Belarus | 10.5 | 6.5 | 45.3 | 37.8 |
| Belize | 9.7 | 6.1 | 61.1 | 23.1 |
| Bosnia and Herzegovina | 5 | 3.2 | 77.7 | 14.1 |
| Botswana | 19.5 | 64.8 | 6.6 | 9.1 |
| Brazil | 7.3 | 4.7 | 68 | 20 |
| Bulgaria | 5.4 | 3.4 | 76.4 | 14.7 |
| China | 6.7 | 4.3 | 70.5 | 18.5 |
| Colombia | 8.4 | 5.2 | 56.5 | 29.8 |
| Costa Rica | 8.8 | 5.7 | 59.4 | 26.1 |
| Cuba | 9.5 | 5.8 | 47.1 | 37.7 |
| Dominica | 6.9 | 4.2 | 63.4 | 25.4 |
| Dominican Republic | 8.6 | 4.4 | 17.1 | 69.9 |
| Ecuador | 5 | 3.1 | 75.8 | 16 |
| Equatorial Guinea | 71.3 | 13.2 | 5.6 | 9.9 |
| Fiji | 5.6 | 3.4 | 70.9 | 20.2 |
| Gabon | 36.7 | 46 | 6.9 | 10.4 |
| Georgia | 9.1 | 5.5 | 46.9 | 38.5 |
| Grenada | 10 | 6.3 | 60.2 | 23.5 |
| Guatemala | 9.5 | 4.7 | 17.8 | 68 |
| Guyana | 24.7 | 9.6 | 17.1 | 48.6 |
| Indonesia | 9.3 | 4.6 | 14 | 72.1 |
| Iran, Islamic Rep. | 11.3 | 7.1 | 56.3 | 25.3 |
| Iraq | 34.6 | 11.8 | 16.2 | 37.4 |
| Jamaica | 20.2 | 63.8 | 6.7 | 9.3 |
| Jordan | 6.3 | 3.9 | 73.3 | 16.5 |
| Kazakhstan | 10.1 | 6 | 40.5 | 43.3 |
| Lebanon | 6.3 | 4 | 72.7 | 17 |
| Libya | 6.4 | 4 | 72.7 | 16.9 |
| Malaysia | 10.4 | 6.4 | 44.2 | 39 |
| Maldives | 6.7 | 4.2 | 71.6 | 17.5 |
| Marshall Islands | 19 | 8.1 | 14.6 | 58.3 |
| Mexico | 7.8 | 5 | 63.3 | 23.9 |
| Montenegro | 8.4 | 5.3 | 59.7 | 26.6 |
| Namibia | 19.3 | 64.7 | 6.8 | 9.2 |
| North Macedonia | 8.6 | 4.9 | 50.3 | 36.3 |
| Paraguay | 9.2 | 4.9 | 26.2 | 59.7 |
| Peru | 8.2 | 4.7 | 45 | 42.1 |
| Russian Federation | 10.7 | 6.3 | 35 | 47.9 |
| Samoa | 14.6 | 6.4 | 13.6 | 65.4 |
| Serbia | 7.4 | 4.3 | 60.1 | 28.2 |
| South Africa | 21.4 | 60.5 | 7.7 | 10.5 |
| St. Lucia | 8.9 | 5.7 | 64.1 | 21.2 |
| St. Vincent and the Grenadines | 11.2 | 7.1 | 56.8 | 24.9 |
| Suriname | 5.1 | 3.2 | 77 | 14.8 |
| Thailand | 8.7 | 4.9 | 40.2 | 46.2 |
| Tonga | 20.7 | 8.5 | 14.6 | 56.2 |
| Turkey | 5.7 | 3.6 | 75.5 | 15.2 |
| Turkmenistan | 33.2 | 11.6 | 16.5 | 38.7 |
| Tuvalu | 5.6 | 3.5 | 73.5 | 17.4 |
| Venezuela, RB | 36 | 11.4 | 14.3 | 38.4 |

**Table H. Model information.** Key variables as determined by Random Forest classifier by MeanDecreaseAccuracy and MeanDecreaseGini.

|  | **Details** |
| --- | --- |
| Type of Random Forest | Classification |
| Number of Trees | 500 |
| Variables Tried per Split | 10 |
| OOB Error Rate | **11.19%** |

**Table I. Confusion Matrix.**

|  | **Cluster 1** | **Cluster 2** | **Cluster 3** | **Cluster 4** | **Class Error** |
| --- | --- | --- | --- | --- | --- |
| **Cluster 1** | 12 | 5 | 0 | 2 | 0.37 |
| **Cluster 2** | 0 | 35 | 1 | 2 | 0.08 |
| **Cluster 3** | 0 | 0 | 51 | 0 | 0.00 |
| **Cluster 4** | 1 | 0 | 4 | 21 | 0.19 |

**Table J. Features and importance.**

| **Feature** | **Mean Decrease Accuracy** | **Mean Decrease Gini** |
| --- | --- | --- |
| International ratio prevention of vertical transmission of HIV subtotal | 11.20872262 | 4.626646119 |
| Total public ratio prevention | 9.836581119 | 3.905677161 |
| International ratio prevention | 10.82884451 | 3.801044853 |
| Life expectancy at birth total years | 9.334201198 | 3.444913813 |
| Year dentists per 10 000 | 7.874718658 | 2.956383012 |
| Under-five mortality rate DHS 2013-2019 | 8.478110187 | 2.829854018 |
| Total public ratio treatment care and support | 9.13940412 | 2.810089427 |
| People using at least basic sanitation services of population | 7.879886587 | 2.744711454 |
| Population ages 0 14 of total population | 8.174831033 | 2.657980234 |
| Maternal mortality ratio modeled estimate per 100 000 live births | 8.084082904 | 2.631160353 |
| HIV prevalence Adults 15 49 | 8.103595783 | 2.348712827 |
| Life expectancy at age 60 female years 2017 | 7.882792298 | 2.261353134 |
| Global Fund resources | 7.162922091 | 2.227916895 |
| Physicians’ density per 1000 population | 6.823976608 | 2.218883064 |
| International ratio Treatment care and support | 7.981239686 | 2.167541015 |
| Total public ratio Prevention of vertical transmission of HIV sub total | 8.997096866 | 2.083965646 |
| Nurse initiated ART allowed for Non pregnant adults | 7.393716599 | 1.936234764 |
| Population ages 65 and above of total population | 8.013593281 | 1.817381797 |
| GDP per capita PPP current international | 6.390978738 | 1.813925711 |
| Population ages 15 64 of total population | 6.518862668 | 1.753766384 |
| Number of people living with HIV all ages | 6.904448888 | 1.365944496 |
| Prevalence of current tobacco use of adults | 5.472406696 | 1.275777273 |
| TOTAL expenditures on HIV USD | 4.297294595 | 1.192813516 |
| People using at least basic sanitation services of population Q1 Q5 | 6.492752154 | 1.183459007 |
| Pharmacists per 10 000 | 6.421288954 | 1.121649742 |
| HIV prevalence sex workers 2019 | 6.153392606 | 1.115929885 |
| Gini index World Bank estimate | 5.33227065 | 1.089211213 |
| Nursing and midwifery personnel density per 1000 population | 6.306066353 | 1.011057474 |
| External health expenditure of current health expenditure | 3.774957256 | 1.008164685 |
| UHC service coverage index World Bank | 4.979249992 | 0.958381856 |
| New HIV infections all ages | 5.405030922 | 0.938186008 |
| Criminalization of sex work | 5.162486378 | 0.898323377 |
| HIV prevalence prisoners 2019 | 3.83095786 | 0.893195838 |
| Domestic general government health expenditure of current health expenditure | 4.078334327 | 0.88407699 |
| Total wealth per capita constant 2014 US 2014 | 3.946374302 | 0.864266481 |
| Hospital beds per 10 000 population | 4.793398887 | 0.835929855 |
| Health facilities delivering integrated services HIV and hepatitis C treatment | 4.965294942 | 0.83423372 |
| Antiretroviral therapy coverage for PMTCT of pregnant women living with HIV | 4.228691849 | 0.814431197 |
| Country has a national policy on HIV self-testing | 5.573211235 | 0.796654748 |
| Life expectancy at age 60 male years 2017 | 3.530740689 | 0.771981889 |
| prevalence diabetes | 4.38950082 | 0.748255525 |
| Government Effectiveness Percentile Rank | 2.619081976 | 0.738572588 |
| Total private ratio: prevention | 3.184837826 | 0.716949201 |
| Control of Corruption Percentile Rank | 2.664866269 | 0.711272191 |
| Health facilities delivering integrated services HIV treatment and care with nutrition support | 4.716737915 | 0.684866488 |
| Out of pocket expenditure of current health expenditure | 4.044601289 | 0.68412288 |
| Tuberculosis case detection rate, all forms | 3.297423022 | 0.614522336 |
| Voice and Accountability Percentile Rank | 2.065732468 | 0.608997954 |
| Domestic general government health expenditure of general government expenditure | 0.033192724 | 0.606979416 |
| Health facilities delivering integrated services HIV testing with child health services | 4.792077651 | 0.602754839 |
| HIV prevalence PWID (2019) | 0.983213668 | 0.593342686 |
| Regulatory Quality Percentile Rank | 3.885598959 | 0.554158404 |
| DTG being introduced as first line ARV regimen where TDF 3TC or FTC EFV not preferred combinations | 4.781458736 | 0.540759944 |
| Catastrophic spending of population spending 10 of income on OOP | 0.014190287 | 0.534790918 |
| Income share bottom 50% (2019) | 2.169285116 | 0.47984607 |
| Antiretroviral therapy coverage of people living with HIV | 0.791301303 | 0.457176672 |
| Rule of Law Percentile Rank | 1.914236331 | 0.45140008 |
| HIV prevalence MSM (2019) | 3.593239627 | 0.447130896 |
| Urban population of total population | 1.689897162 | 0.446045673 |
| Received all 8 basic vaccinations DHS (2013-2019) | 1.696270382 | 0.444029098 |
| Political Stability and Absence of Violence Terrorism Percentile Rank | 2.171198994 | 0.431366493 |
| Immunization DPT of children ages 12-23 months | 3.763632977 | 0.430830193 |
| Children with ARI for whom advice or treatment was sought from a health facility or provider DHS 2013 19 | 2.477902594 | 0.411494427 |
| Dept to GDP ratio (2015) | 0.723986555 | 0.404238597 |
| COVID case fatality ratio | 1.929360505 | 0.39850189 |
| Health facilities delivering integrated services Violence screening and mitigation with HIV services | 3.459049078 | 0.396001041 |
| Health facilities delivering integrated services HIV treatment and care with child health services | 2.696458985 | 0.388943936 |
| GDP cap growth 2000 2018 WHO | 2.416675466 | 0.379892213 |
| Current health expenditure of GDP | -0.856756538 | 0.364942087 |
| Tuberculosis treatment success rate of new cases | 1.455796814 | 0.354484461 |
| Implementation status of same day start of ART | 2.685206558 | 0.347247186 |
| Income share Top10 (2019) | 1.776425032 | 0.346598336 |
| Unmet need for family planning DHS (2013-2019) | 1.53554718 | 0.344122144 |
| X2020 prevention targets for MSM of MSM reached by HIV prevention services | 1.534768696 | 0.333939113 |
| HIV expenditure on key populations aggregate of all HIV exp in year | -0.371545057 | 0.324845437 |
| Criminalization of same sex sexual acts | 0.256882986 | 0.321067581 |
| WHO recommendation on oral PrEP adopted in country s national guidelines | 3.127314923 | 0.314271562 |
| Tuberculosis effective treatment coverage | -0.202139137 | 0.310488968 |
| X2020 prevention targets for sex workers of sex workers reached by HIV prevention services | 0.161923412 | 0.303839 |
| Health facilities delivering integrated services HIV counselling and testing and chronic NCDs | 2.473111313 | 0.299858615 |
| Total private ratio for treatment care and support | 1.681665546 | 0.295690433 |
| Hypertensive n | 0.797944636 | 0.28401111 |
| Health facilities delivering integrated services HIV testing and counselling with cervical cancer screening and treatment | 3.300508024 | 0.278897661 |
| Health facilities delivering integrated services HIV treatment and care with SRH | 1.527999843 | 0.264679695 |
| Health facilities delivering integrated services ART and general outpatient care | 2.123086034 | 0.236477755 |
| Implementation of ART provision in community settings for people stable on ART | 1.889177802 | 0.210903889 |
| ART provided in community settings for people stable on ART | 0.409012657 | 0.1980602 |
| Laws requiring parental consent for adolescents to access HIV testing | 1.059350041 | 0.177730656 |
| Self-testing implemented in countries with a national self-testing policy | 2.06587931 | 0.174462448 |
| Laws criminalizing transmission of non-disclosure of or exposure to HIV transmission | 2.997585923 | 0.152861303 |
| Health facilities delivering integrated services HIV and harm reduction services | 1.660078053 | 0.122631574 |
| Health facilities delivering integrated services HIV counselling and testing and general outpatient care | 1.027197071 | 0.109363114 |
| Health facilities delivering integrated services Cervical cancer screening with HIV services | 2.041470823 | 0.10464918 |
| Health facilities delivering integrated services ART and chronic NCDs | 1.602853528 | 0.090186303 |
| User fees PHC, HIV | -0.655517208 | 0.088173813 |
| Health facilities delivering integrated services ART and TB treatment | -0.582889681 | 0.076200413 |
| COVID 19 Debt Service Suspension Initiative DSSI eligible | -0.25417267 | 0.06828407 |
| WHO 2015 policy update on use of LF LAM for diagnosis and screening of active TB in people living with HIV adopted | -0.006416711 | 0.056348244 |
| Health facilities delivering integrated services HIV counselling and testing with SRH | 2.163329552 | 0.056283289 |
| Health facilities delivering integrated services HIV counselling and testing with TB services | 0.068233738 | 0.048927324 |
| Health facilities delivering integrated services TB screening in HIV services | 2.004112234 | 0.044555005 |
| Assisted HIV partner notification included in national policy | 1.001001503 | 0.043362637 |
| Budget trend 20 21 | 1.032777772 | 0.042846436 |
| Human rights monitoring and enforcement mechanisms Independent functional institution for promotion and protection of human rights | -0.262759018 | 0.039299136 |
| Education policies on life skills-based HIV and sexuality education primary school | 1.836899641 | 0.037757622 |
| Method to de duplicate key data i e unique identifiers | 0.509118512 | 0.037279509 |
| Any laws provisions specifying protections based on grounds of sexual orientation | 1.284661161 | 0.03525432 |
| Country has a national plan for eliminating HIV MTCT | 1.41672858 | 0.032021081 |
| National policy on routine viral load testing for adults and adolescents | -0.101792993 | 0.031601039 |
| Health facilities delivering integrated services PMTCT with antenatal care maternal and child health | -1.422339342 | 0.026418211 |
| Possible to start ART on the same day as HIV diagnosis | 1.414161068 | 0.02348537 |
| HIV case surveillance system in the country | 1.001001503 | 0.020835736 |
| Universal health insurance scheme in country | -1.001001503 | 0.0195 |
| National plan strategy addressing gender-based violence against women that includes HIV | -1.416470149 | 0.018694056 |
| Recommended ART CD4 initiation threshold in adults and adolescents | 0 | 0.018600464 |
| Any restrictions to registration operation of civil society CBOs affecting HIV service delivery | 0.028278639 | 0.017345455 |
| Compulsory detention for people who use drugs applied | 1.001001503 | 0.012799346 |
| Explicit supportive reference to harm reduction in national policies | -1.001001503 | 0.006207358 |
| Benefits package for universal health insurance scheme includes ARVs for countries with such a scheme | -1.001001503 | 0.005666667 |
| Routine user fees or charges for services at public health facilities | -1.001001503 | 0.003427322 |
