# Supplementary material for: Using country-level variables to discover country clusters beyond traditional health policy and performance metrics: An unsupervised machine learning approach for HIV healthcare delivery and financing
Source: PLOS Glob Public Health. 2025 Dec 2;5(12):e0004583. doi: 10.1371/journal.pgph.0004583 (PMC12671754; doi:10.1371/journal.pgph.0004583)
Supplement: Attachment — Submitted filename: Response letter.docx [file pgph.0004583.s002.docx]

**Response letter**

**PGPH-D-25-00288
Identifying clusters of countries with similar progression towards sustainable HIV service delivery and financing: an unsupervised machine learning approach**

**PLOS Global Public Health**

To the Editors,

Please find enclosed our revised manuscript and our response letter. The title of the manuscript has been revised to “**Using country-level variables to discover country clusters beyond traditional health policy and performance metrics: An unsupervised machine learning approach for HIV healthcare delivery and financing**” to reflect the added value of our ML approach for policymaking, as noted by one of the reviewers.

We have carefully considered and incorporated the reviewers’ suggestions. The most significant changes include:

1) Revisions to the Introduction to reflect recent developments, particularly funding cuts and post-COVID-19 recovery.
2) A strengthened rationale for the selected machine learning methods, along with an exploration of alternative methods for validation and comparison.
3) Expanded Discussion section, including:
   a. Feedback from various policymakers on the clustering results and the practical utility of our approach.
   b. A more detailed explanation of how this method can be applied and further developed in future work.

We thank you for the opportunity to resubmit a revised version of our manuscript. We have provided a version of the manuscript in which all changes as compared to the originally submitted version are highlighted in track changes, on top of a clean version of the revised manuscript.

We look forward to receiving your response.

Sincerely,

Dr. Caroline Bulstra

*On behalf of all authors*

--

Dear Dr. Bulstra,

Thank you for submitting your manuscript to PLOS Global Public Health. After careful consideration, we feel that it has merit but does not fully meet PLOS Global Public Health’s publication criteria as it currently stands. Therefore, we invite you to submit a revised version of the manuscript that addresses the points raised during the review process.

--

Reviewers' comments:

Reviewer's Responses to Questions

**Comments to the Author**

1. Does this manuscript meet PLOS Global Public Health’s [publication criteria](https://urldefense.proofpoint.com/v2/url?u=https-3A__track.editorialmanager.com_CL0_https-3A-252F-252Fjournals.plos.org-252Fglobalpublichealth-252Fs-252Fcriteria-2Dfor-2Dpublication_1_010f01978344381e-2De4cd13db-2D4c2b-2D47a9-2D8674-2Dd4c119db52cc-2D000000_LZKQDHf6VJslkOpBMQjTKNNKQOERijsciqJe5wmMyKo-3D216&d=DwMFaQ&c=WO-RGvefibhHBZq3fL85hQ&r=3pCKIjVLg5bc6Tyf4cWJcU5C2ty2JpM-cmirhamSoMk&m=VsY8Xv5VVkz4RIS3XhJxFjA7Eztz-dcYcMrlReFEXIhF4MKYl03iRtg76Si-x0v6&s=0WSnE_Y-s3Of2idAXBbi_hrWT9QV4P5_OPwsaBUWpJA&e=)? Is the manuscript technically sound, and do the data support the conclusions? The manuscript must describe methodologically and ethically rigorous research with conclusions that are appropriately drawn based on the data presented.

Reviewer #1: Yes

Reviewer #2: Yes

2. Has the statistical analysis been performed appropriately and rigorously?

Reviewer #1: Yes

Reviewer #2: Yes

3. Have the authors made all data underlying the findings in their manuscript fully available (please refer to the Data Availability Statement at the start of the manuscript PDF file)?

The [PLOS Data policy](https://urldefense.proofpoint.com/v2/url?u=https-3A__track.editorialmanager.com_CL0_https-3A-252F-252Fjournals.plos.org-252Fglobalpublichealth-252Fs-252Fdata-2Davailability_1_010f01978344381e-2De4cd13db-2D4c2b-2D47a9-2D8674-2Dd4c119db52cc-2D000000_w0W2kHxFwDG4MKAwKA-2DhFi3czRZmWWd3oYMx1xrSFPs-3D216&d=DwMFaQ&c=WO-RGvefibhHBZq3fL85hQ&r=3pCKIjVLg5bc6Tyf4cWJcU5C2ty2JpM-cmirhamSoMk&m=VsY8Xv5VVkz4RIS3XhJxFjA7Eztz-dcYcMrlReFEXIhF4MKYl03iRtg76Si-x0v6&s=dM8Hy9SZs8Njv-aVt8u2OeX4DEhGQVLv0SYcuRaXs14&e=) requires authors to make all data underlying the findings described in their manuscript fully available without restriction, with rare exception. The data should be provided as part of the manuscript or its supporting information or deposited to a public repository. For example, in addition to summary statistics, the data points behind means, medians and variance measures should be available. If there are restrictions on publicly sharing data—e.g. participant privacy or use of data from a third party—those must be specified.

Reviewer #1: Yes

Reviewer #2: Yes

4. Is the manuscript presented in an intelligible fashion and written in standard English?

PLOS Global Public Health does not copyedit accepted manuscripts, so the language in submitted articles must be clear, correct, and unambiguous. Any typographical or grammatical errors should be corrected at revision, so please note any specific errors here.

Reviewer #1: Yes

Reviewer #2: Yes

**Review Comments to the Author**

Please use the space provided to explain your answers to the questions above. You may also include additional comments for the author, including concerns about dual publication, research ethics, or publication ethics. (Please upload your review as an attachment if it exceeds 20,000 characters)

**Reviewer #1:**

*We thank Reviewer #1 for their detailed and constructive feedback, which helped us improve both the methodology and clarity of our manuscript. We have made several major revisions in response, which we outline below.* *Importantly, the groupings identified in the initial analysis remain broadly consistent albeit more robust after the revisions, and our overall reasoning and discussion are unaffected.*

1. Why chose K-means clustering?

*The dataset used in this study is relatively small and low-dimensional, which informed our decision to use interpretable, distance-based clustering methods rather than more complex or highly stochastic algorithms that may be less suitable in this context. Therefore, we initially chose K-means clustering and compared with hierarchical and Partitioning Around Medoids (PAM) in the appendices. However, based on the comments of the reviewers we have expanded and included partitional, hierarchical, spectral, and density-based algorithms. K-means was not included in the current analyses, as we selected PAM to represent partitional clustering given its robustness to outliers and its better alignment with the other methodological changes (including the use of UMAP). The overall interpretation and discussion remain unaffected.*

*The new Figure 2 shows the two-dimensional UMAP plots. Each point represents a country positioned along the first and second UMAP dimensions. The spatial distribution reflects similarity across all input indicators, with clusters appearing as groups of points that are closer together in this reduced-dimensional space. Algorithms we included were: PAM = partitioning around medoids, HC = hierarchical, DBSCAN = Density-based spatial clustering of applications with noise.*

*The process of- and rationale for selecting the different algorithms has been updated in the* ***Methods section*** *of the manuscript.*

2. Why chose PCA? Scree plot indicates that the variables are not in relative high correlation, then what the purpose of PCA? PCA could additionally reduce the interpretation, though. 25% of the total variance explained is too low for PCA even though authors claimed their purpose was to highlight groups. At least a sensitivity analysis of using more PCs that count for more variance (e.g. 80%) is needed.

*We thank the reviewer for these comments. Our initial choice for PCA was motivated by its common use as an exploratory tool to visualize potential groupings in multivariate data. However, in our dataset the correlation structure between variables was limited, leading to a relatively low proportion of variance explained by the first two principal components. This restricted the interpretability and usefulness of the PCA plots.*

*To address this, we have revised our analysis and replaced PCA with Uniform Manifold Approximation and Projection (UMAP), a non-linear dimensionality reduction technique that is better suited for mixed data structures such as ours. UMAP preserves both local and global structure and provides clearer visualization of groupings, thereby avoiding the interpretational drawbacks noted by the reviewer.*

3. How can you know your clusters are (not) mainly determined by general socioeconomic factors or general health level instead of HIV related factors. For example, one could just use K-means clustering general socioeconomic factors that results in similar 4 clusters and post hoc compare some HIV indicators. Post-hoc comparison?

*Indeed, it is important to know whether clusters are not mainly determined by general socioeconomic factors or general health level instead of HIV related factors. To address the concern that clusters might be driven by only a subset of variables, we additionally assess variable importance using random forests and calculated Cramer’s V coefficients to compare clusters against existing typologies. This triangulation provides reassurance that clusters reflect multidimensional profiles rather than being determined by single dominant variables.*

*Below is the revised section outlining the steps to address the raised concerns (page 21, lines 10-17):*

*“Random Forest, an ensemble of decision trees, was applied post-hoc to identify the variables most important for distinguishing clusters, out of the box error (OOB) was used to estimate the prediction error. Variable importance was quantified using* *MeanDecreaseAccuracy, which measures how much model accuracy decreases when a variable is excluded, and MeanDecreaseGini, which reflects how much a variable contributes to reducing node impurity across the trees (27). The top 20 key input indicators were then selected based on the Random Forest results. We calculated correlation coefficients (Cramer’s V Coefficient (φc)) to assess if clusters represent groupings that are recognizably different from existing typologies by world region and HIV/AIDS epidemic type.”*

*Furthermore, we have now post-hoc applied Random Forest, an ensemble of decision trees, to identify the variables most important for distinguishing clusters.*

*The Random Forest results and the correlation coefficients were included in the main text (page 23, from line 17):*

*“The Random Forest classifier achieved good performance in predicting the PAM clusters (OOB error 11%). Cluster 3 was classified perfectly, while clusters 1 and 4 showed some misclassification, mainly with cluster 2. The Random Forest classifier also identified the key variables driving cluster assignment. The most influential variables spanned the subject domains Universal Health Coverage (dentists density, physician density, nurse-initiated ART access), HIV/AIDS epidemic (adult HIV prevalence), healthcare financing (international and public funding ratios for HIV e.g., for prevention, treatment and vertical transmission, Global Fund resources), demographic and socioeconomic factors (population age structure, access to basic sanitation, per capita GDP), and health and mortality outcomes (life expectancy at birth and age 60, maternal and under-five mortality rates) (****Table 2****,* ***Appendix S4****). Indicators from the subject domains 'HIV response' and 'Legislation about human rights/the HIV response' were not included among the top 20 variables but showed measurable contributions to cluster assignment.”*

4. Figure 1A needs more explanation. Readers may not understand the meaning of it.

*We have now revised the figure to include all explored algorithms. We have updated the caption of this and other figures to include a more detailed explanation of the visuals and methods applied (page 30).*

*“****Fig 1. Silhouette scores for each clustering algorithm.*** *The silhouette score represents how well an observation fits within its assigned cluster compared to neighbouring clusters, with higher values indicating better-defined and more cohesive clusters. PAM = partitioning around medoids, HC = hierarchical, DBSCAN = density-based spatial clustering of applications with noise.”*

*Updated Figure 1:*

*We have also added more detailed explanations of the applied algorithms to the Methods section of the manuscript (starting from page 20, line 12):*

*“We used unsupervised machine learning algorithms to cluster country-level data, using several approaches including partitional (Partitioning Around Medoids (PAM)), hierarchical, spectral, and density-based (DBSCAN) methods (25). As a pre-processing step, Gower distances were computed to handle mixed data types, followed by Uniform Manifold Approximation and Projection (UMAP), a non-linear dimensionality reduction technique using default parameters (15 neighbours and a minimum distance of 0.1) (26).*

*PAM clustering was applied using the Euclidean distance metric, with default initialization and iterative optimization until convergence. We used the Silhouette Index (with values above 0.50 considered acceptable based on reference standard) and the Gap Statistic (with an substantial increase indicating better clustering) to assess the optimal number of clusters for PAM, iterating between solutions with 2:5 clusters [13]. Cluster stability was evaluated using bootstrapping with mean Jaccard bootstrap values across 1000 samples (>0.70 considered stable).*

*Hierarchical (HC) and spectral clustering were applied specifying four clusters, and DBSCAN was run with eps = 0.5 and minPts = 5, resulting in 4 clusters and no points unclustered. The Adjusted Rand Index (ARI) was used to compare consistency across all four clustering algorithms. An ARI above 0.70 indicates strong consistency, 0.50–0.70 suggests moderate agreement, and below 0.50 indicates poor consistency [14,15]. We then used silhouette plots, 2D UMAP plots and country maps to visualize country typologies across algorithms.*

*The most robust and easy-to-interpret cluster solution was then chosen and used for downstream analyses to aid interpretation of clusters. Fuzzy clustering was additionally used to examine membership probabilities at cluster boundaries, which were visualized using country maps (20).*

*Random Forest, an ensemble of decision trees, was applied post-hoc to identify the variables most important for distinguishing clusters, out of the box error (OOB) was used to estimate the prediction error. Variable importance was quantified using MeanDecreaseAccuracy, which measures how much model accuracy decreases when a variable is excluded, and MeanDecreaseGini, which reflects how much a variable contributes to reducing node impurity across the trees (27). The top 20 key input indicators were then selected based on the Random Forest results. We calculated correlation coefficients (Cramer’s V Coefficient (φc)) to assess if clusters represent groupings that are recognizably different from existing typologies by world region and HIV/AIDS epidemic type.*

*Finally, each cluster was summarized into a policy‑interpretable “profile name” by extracting original mean or median values of key variables identified by the Random Forest. These profiles were then refined by consensus within our multidisciplinary team, incorporating both HIV/AIDS prevalence and region alongside new insights from this study.”*

**Reviewer #2:**

Reviewer #2: Major Concerns

*We thank the Reviewer #2 for their constructive feedback, which helped us further improve our analysis and interpretation. We have made several major changes in response, which we outline below. Importantly, the groupings identified in the initial analysis remain broadly consistent also after further optimizing the applied ML algorithms.*

1. Over-reliance on K-means clustering

Although the authors mention K-means, hierarchical clustering, and Fuzzy K-means, the core analysis depends solely on K-means. This approach is sensitive to initial centroid selection and Euclidean distance in high-dimensional data. The study lacks comparative experiments (e.g., DBSCAN, Gaussian Mixture Models, Spectral Clustering) to improve robustness.

*The dataset used in this study is relatively small and low-dimensional, which informed our decision to use interpretable, distance-based clustering methods rather than more complex or highly stochastic algorithms that may be less suitable in this context. Therefore, we initially chose K-means clustering and compared it with hierarchical clustering and Partitioning Around Medoids (PAM) in the appendices. Based on reviewer comments, we expanded the analysis to include partitional, hierarchical, spectral, and density-based algorithms. In the revised manuscript, we now more clearly emphasize the results from all clustering approaches. Hierarchical and spectral clustering produced broadly consistent cluster assignments with PAM, as reflected in high Adjusted Rand Index (ARI) values, supporting the robustness of our findings. As expected, DBSCAN performed less well on our dataset: due to the small sample size and inter-country distances, it often produced either a single cluster containing most observations or many small clusters, even across a wide range of parameter values. This behavior aligns with known limitations of density-based methods in small-sample settings. These expanded analyses, including silhouette scores, ARI comparisons, and cluster stability assessments, are presented in the Data Analysis and Results section of the revised manuscript. K-means was not included in the current analyses, as we selected PAM to represent partitional clustering given its robustness to outliers and its better alignment with the other methodological changes (including the use of UMAP). The overall interpretation and discussion remain unaffected.*

*The Data Analysis Section presents the revised analysis undertaken (starting on page 20, line 12):*

*“We used unsupervised ML algorithms to cluster country-level data, using several approaches including partitional (**Partitioning Around Medoids; PAM), hierarchical, spectral, and density-based (DBSCAN) methods (25). As a pre-processing step, Gower distances were computed to handle mixed data types, followed by Uniform Manifold Approximation and Projection (UMAP), a non-linear dimensionality reduction technique using default parameters (15 neighbours and a minimum distance of 0.1) (26).*

*PAM clustering was applied using the Euclidean distance metric, with default initialization and iterative optimization until convergence. We used the Silhouette Index (with values above 0.50 considered acceptable based on reference standard) and the Gap Statistic (with an substantial increase indicating better clustering) to assess the optimal number of clusters for PAM, iterating between solutions with 2:5 clusters [13]. Cluster stability was evaluated using bootstrapping with mean Jaccard bootstrap values across 1000 samples (>0.70 considered stable).*

*Hierarchical (HC) and spectral clustering were applied specifying four clusters, and DBSCAN was run with eps = 0.5 and minPts = 5, resulting in 4 clusters and no points unclustered. The Adjusted Rand Index (ARI) was used to compare consistency across all four clustering algorithms. An ARI above 0.70 indicates strong consistency, 0.50–0.70 suggests moderate agreement, and below 0.50 indicates poor consistency [14,15]. We then used silhouette plots, 2D UMAP plots and country maps to visualize country typologies across algorithms.”*

*The result section presents the results of the analysis (page 10, lines 2-14):*

*“Using PAM clustering, we found four clusters (k = 4) solution robust and interpretable, as the silhouette score remained above 0.50, with acceptable scores for k = 2 (0.671), k = 3 (0.583), and k = 4 (0.527), coupled with a favourable visual assessment of the Gap Statistic metrics (****Fig 1,*** ***S2*** ***Appendix****). Bootstrap validation showed the following mean Jaccard bootstrap values: cluster 1 (0.779), cluster 2 (0.928), cluster 3 (0.934), and cluster 4 (0.887).*

*Comparison between the four clustering algorithms showed that PAM and hierarchical clustering (HC) had very similar average silhouette scores (0.53 and 0.53, respectively), indicating good cluster separation, while spectral (0.46) and DBSCAN (0.44) showed slightly lower, but still acceptable, silhouette values.* *Adjusted Rand Index (ARI) values demonstrated strong agreement between PAM and HC (0.929), as well as between PAM and spectral clustering (0.835). In contrast, agreement with DBSCAN was lower, particularly with PAM (0.710) and HC (0.672), indicating moderate consistency (****S2 Appendix****).”*

2. Insufficient variance explained by PCA

The authors retain only three principal components (explaining ~25% of variance), which is unusually low for social science research. While they justify this as "highlighting groups," such low information retention may distort clustering. They should explore retaining more components (did approach of 70%; shows cluster 1 and cluster 2 and almost the same, does show more variation in cluster 3 and 4) or using nonlinear methods (e.g., t-SNE, UMAP) (only for visualizing or also for presenting the data?

*We thank the reviewer for this valuable suggestion. We initially applied PCA as an exploratory tool to assess potential groupings in the data, a common first step in multivariate analyses. However, in our dataset the limited correlation structure between variables resulted in a low proportion of variance explained by the first two components, restricting interpretability. We have therefore revised our analysis and replaced PCA with Uniform Manifold Approximation and Projection (UMAP), a non-linear dimensionality reduction technique that is particularly suited for mixed data structures. UMAP better preserves both local and global structure and provides clearer visualization of groupings, addressing the drawbacks noted by the reviewer.*

3. No stability or statistical significance test

Beyond Silhouette and Adjusted Rand Index, the study does not assess cluster stability (e.g., bootstrapping, Cluster Stability Analysis, Gap Statistic). Without evaluating robustness to sample variations, the reliability and reproducibility of results remain unclear.

*We agree with the reviewer and therefore added the Gap Statistic to guide the number of clusters and a bootstrap-based stability assessment (Jaccard index). These analyses confirmed that the chosen four-cluster solution was robust, with consistent support across the different metrics. The silhouette scores and Gap Statistic indicated a well-fitting and interpretable solution, and the bootstrap results demonstrated high stability of all clusters. Together, these findings provide confidence in the reliability and reproducibility of our clustering results.*

4. Lack of empirical or policy validation

The authors claim policy relevance but do not apply clusters to real-world HIV program comparisons or link them to independent data (e.g., health expenditure, outbreak control). The work remains at a "proof-of-concept" stage with weak policy connections.

*We appreciate this comment and agree that our work is currently best positioned as a proof-of-concept. Clusters were validated and discussed with policymakers from UNAIDS, WHO and other institutions, outside of the author list. These stakeholders signaled that the approach is useful as an exploratory tool to complement current policy procedures. Specifically, they highlighted that clustering based on multiple dimensions—beyond geography, burden of disease, or funding source—can help identify tailored strategies and foster cross-country learning. For now, we believe the current analysis offers a valuable and interpretable foundation for both research and policy dialogue. We have updated sections throughout the Introduction to reflect this idea.*

*Furthermore, we have added this to the discussion section of the manuscript (page 27, lines 15-20):*

*“The added value of ML lies in its capacity to incorporate multi-criteria data—such as legal environments for key populations, integration of HIV services into UHC, and health spending efficiency—which could yield typologies directly applicable to policymaking. In the future, moving towards the cascade targets, countries that have already achieved the* *95–95–95 targets (1), such as Botswana and Rwanda, may offer lessons to others within their cluster that face similar system constraints but are at an earlier stage of transition.”*

5. Unclear handling of high-dimensional heterogeneous data

The study integrates 120 diverse variables (institutional, binary, continuous) using FAMD but lacks transparency in weighting, scaling, and outlier control. This may affect clustering quality and fairness.

*We have now replaced the original FAMD approach with Gower distance-based clustering to better handle our mixed data types (continuous, binary, categorical). Dimensionality was then reduced using Uniform Manifold Approximation and Projection (UMAP), a non-linear technique that preserves both local and global structure, followed by Partitioning Around Medoids (PAM) clustering. This approach improves interpretability, visual separation of clusters, and robustness compared with linear methods like FAMD, particularly for small, heterogeneous country-level datasets.*

Minor Comments

1-Missing data and imputation accuracy unreported

Provide missing rates per variable category and evaluate imputation errors (e.g., RMSE or comparison with complete cases) to avoid bias.

*We appreciate the suggestion by the reviewer to provide missing rates per variable category and evaluate imputation errors. We have now analyzed the missingness per variable in the dataset and added a table to the appendix detailing the missing data rate for each variable (please refer to S1_file for the table).*

2-Insufficient analysis of Fuzzy Clustering results

The fuzzy clustering map is provided, but membership probabilities and mixed-boundary countries are not discussed in depth.

*We thank the reviewer for this comment. We have clarified the interpretation of the fuzzy clustering results, both in the figure and in the Results text, with additional explanation of membership probabilities and mixed-boundary countries.*

3-No external or temporal validation

All data are post-2017. Consider a hold-out validation set or temporal splits to test generalizability.

*Our study employs a cross-sectional design aimed at creating a comprehensive and up-to-date snapshot of each country using the most recent available data. We prioritized data from 2017 onwards, but in instances where data after 2017 was not available, we expanded our timeframe to include data back to 2000. This approach was taken to ensure a robust dataset with maximum inclusion of variables and countries. Therefore, the primary variation in our dataset is across countries rather than over time. Implementing temporal hold-outs would require consistent pre-2017 panels for many indicators, which are unavailable.*

4-Undefined evaluation thresholds

The Silhouette score (0.35) lacks a reference standard (e.g., >0.5 for good clustering). Clarify thresholds and discuss low-confidence clusters.

*We have now clarified the interpretation of Silhouette scores in the Methods section. In line with standard practice, scores above 0.50 indicate well-defined clusters. In the revised Results, we no longer present clusters with Silhouette scores below 0.50, ensuring that all reported clusters represent reasonably cohesive groupings.*

5-No feature importance analysis

Unsupervised feature importance (e.g., Random Forest-based) could identify key variables for policy interpretation.

*We thank the reviewer for this helpful suggestion. We have now applied Random Forest, an ensemble of decision trees, to identify the variables most important for distinguishing clusters (page 12).*

*“Random forest models identified the key variables driving cluster assignment. The most influential variables spanned the subject domains Universal Health Coverage (dentists density, physician density, nurse-initiated ART access), HIV/AIDS epidemic (adult HIV prevalence), healthcare financing (international and public funding ratios for HIV e.g., for prevention, treatment and vertical transmission, Global Fund resources), demographic and socioeconomic factors (population age structure, access to basic sanitation, per capita GDP), and health and mortality outcomes (life expectancy at birth and age 60, maternal and under-five mortality rates) (Appendix S4).”*

6-Limited comparison with prior work

The discussion does not benchmark against existing WHO/UNAIDS classifications or models (e.g., Kavanagh et al.), reducing persuasiveness.

*We thank the reviewer for pointing to these and other publications. Although we references the most relevant publications in the earlier version of our manuscript, we now highlight the utilized approaches in the Discussion section and have added a comparison of the different ML methodologies (Discussion section, page 27, starting at line 17):*

*“Kavanagh et al. (2020) demonstrated , through the work with the HIV Policy Lab, that HIV-related law and policy environments vary widely across countries and regions, underscoring the limitations of relying solely on geographic or epidemic classifications[17]. Allel et al. (2022) further showed how efficiency in HIV/AIDS spending differs significantly across 78 countries, highlighting the value of multi-dimensional approaches that integrate financing and service delivery factors. Together, these studies support the use of unsupervised methods to generate typologies that reflect not only epidemiological and regional patterns but also legal, financial, and systemic dimensions of the HIV response.”*

7-Unaddressed within-cluster heterogeneity

Countries like "South Africa vs. Namibia" or "China vs. Mexico" may differ significantly in policy and epidemic management. Explain their grouping and potential misclustering.

*We thank the reviewer for this important comment. Indeed, some of the countries within the same cluster might vary considerably with regard to current policy and epidemic management. However, the clustering reflects similarities across the selected indicators (e.g., epidemic type, financing, and service coverage), while inevitably not capturing all contextual differences. We have clarified this in the revised discussion, emphasizing that such within-cluster heterogeneity should not be seen as a limitation only, but also as an opportunity to generate insights into how countries with broadly similar profiles address challenges differently. For example, South Africa and Namibia share financing and coverage characteristics but diverge in policy and epidemic management, offering lessons for comparative analysis. Similarly, China and Mexico cluster together on systemic factors despite different policy and epidemic contexts.*

*We now highlight this in the revised Discussion section (page 27, lines 5-14):*

*“Compared to existing UNAIDS and WHO classifications, which often group countries by geographic region or epidemic burden (14, 15), our ML-derived clusters highlight cross-regional similarities and divergences. For example, while sub-Saharan Africa largely groups together under both approaches, our analysis unexpectedly clusters China with Latin America, and identifies outliers such as Sri Lanka and Papua New Guinea—patterns not visible in standard typologies. This illustrates how ML approaches can supplement existing classification models by dynamically incorporating financing and service delivery dimensions. For instance, Zimbabwe and the Caribbean both fall under different regions and face different local epidemics, but both have extensive community healthcare worker programs and currently learning from each other to further strengthen their local health responses (30).”*

6. PLOS authors have the option to publish the peer review history of their article ([what does this mean?](https://urldefense.proofpoint.com/v2/url?u=https-3A__track.editorialmanager.com_CL0_https-3A-252F-252Fjournals.plos.org-252Fglobalpublichealth-252Fs-252Feditorial-2Dand-2Dpeer-2Dreview-2Dprocess-2523loc-2Dpeer-2Dreview-2Dhistory_1_010f01978344381e-2De4cd13db-2D4c2b-2D47a9-2D8674-2Dd4c119db52cc-2D000000_cWsaBL42k-5FQMA8X6zKp-5FTzh-2Dtd7aVtpdyqMoEWHMnqA-3D216&d=DwMFaQ&c=WO-RGvefibhHBZq3fL85hQ&r=3pCKIjVLg5bc6Tyf4cWJcU5C2ty2JpM-cmirhamSoMk&m=VsY8Xv5VVkz4RIS3XhJxFjA7Eztz-dcYcMrlReFEXIhF4MKYl03iRtg76Si-x0v6&s=f4RdZ-g6x07fTKU9BbUf8xxhumvIK-LOR5Xo2Ta-PQI&e=)). If published, this will include your full peer review and any attached files.


**Do you want your identity to be public for this peer review?** If you choose “no”, your identity will remain anonymous but your review may still be made public.

For information about this choice, including consent withdrawal, please see our [Privacy Policy](https://urldefense.proofpoint.com/v2/url?u=https-3A__track.editorialmanager.com_CL0_https-3A-252F-252Fwww.plos.org-252Fprivacy-2Dpolicy_1_010f01978344381e-2De4cd13db-2D4c2b-2D47a9-2D8674-2Dd4c119db52cc-2D000000_1tgTxb3f7UycZNudkVg0vwhjT61CsVjEZ38Vey6ohyM-3D216&d=DwMFaQ&c=WO-RGvefibhHBZq3fL85hQ&r=3pCKIjVLg5bc6Tyf4cWJcU5C2ty2JpM-cmirhamSoMk&m=VsY8Xv5VVkz4RIS3XhJxFjA7Eztz-dcYcMrlReFEXIhF4MKYl03iRtg76Si-x0v6&s=8K1sZkOQfR1nlrSwhh-dgMYLAOkIQGzYaA_5OJuGpPs&e=).

Reviewer #1: No

Reviewer #2: No

[NOTE: If reviewer comments were submitted as an attachment file, they will be attached to this email and accessible via the submission site. Please log into your account, locate the manuscript record, and check for the action link "View Attachments". If this link does not appear, there are no attachment files.]

While revising your submission, please upload your figure files to the Preflight Analysis and Conversion Engine (PACE) digital diagnostic tool, [https://pacev2.apexcovantage.com/](https://urldefense.proofpoint.com/v2/url?u=https-3A__pacev2.apexcovantage.com_&d=DwMFaQ&c=WO-RGvefibhHBZq3fL85hQ&r=3pCKIjVLg5bc6Tyf4cWJcU5C2ty2JpM-cmirhamSoMk&m=3eRKr7gexsmq8FI0txrAzP5RzI0wbkXIDF7NQYfBcg8hVMDgm8hzxYOMx7e7fu0S&s=8SnqBuXi6pEsz24JblynIovEKEZVKxLXRSZQWNvaUV4&e=). PACE helps ensure that figures meet PLOS requirements. To use PACE, you must first register as a user. Registration is free. Then, login and navigate to the UPLOAD tab, where you will find detailed instructions on how to use the tool. If you encounter any issues or have any questions when using PACE, please email PLOS at [figures@plos.org](mailto:figures@plos.org). Please note that Supporting Information files do not need this step.

*In compliance with data protection regulations, you may request that we remove your personal registration details at any time.* [*(Remove my information/details)*](https://urldefense.proofpoint.com/v2/url?u=https-3A__track.editorialmanager.com_CL0_https-3A-252F-252Fwww.editorialmanager.com-252Fpgph-252Flogin.asp-253Fa-3Dr_1_010f01978344381e-2De4cd13db-2D4c2b-2D47a9-2D8674-2Dd4c119db52cc-2D000000_C9j5lK-5F8h6GrUAli7B3dUM9DVdZah20TG74CBDgg-2DWQ-3D216&d=DwMFaQ&c=WO-RGvefibhHBZq3fL85hQ&r=3pCKIjVLg5bc6Tyf4cWJcU5C2ty2JpM-cmirhamSoMk&m=VsY8Xv5VVkz4RIS3XhJxFjA7Eztz-dcYcMrlReFEXIhF4MKYl03iRtg76Si-x0v6&s=0bLIgjDvkm85weIGlHHA3V6TSd_LiP-eN8BwsZR4FFk&e=)*. Please contact the publication office if you have any questions.*
